# A Case Report of Hyperostosis Frontalis Interna

**DOI:** 10.7759/cureus.24967

**Published:** 2022-05-13

**Authors:** Luis A Alvarez, William Corrigan, Seth Gardner

**Affiliations:** 1 College of Osteopathic Medicine, Lake Erie College of Osteopathic Medicine Bradenton, Bradenton, USA

**Keywords:** estrogen, bone growth, postmenopause, hyperostosis frontalis interna, class c hfi, hfi

## Abstract

A routine dissection of an 89-year-old female cadaver who had died of cardiopulmonary arrest revealed a unique case of hyperostosis frontalis interna (HFI). Multiple layers of spongy bone growth deep to the internal table were coupled with asymmetrical nodular growths. Slight superior sagittal sinus growth was also noted, which is atypical of this condition. Additionally, this cadaver represents one of the rarer and more severe forms of HFI, class C. A clear consensus on whether HFI presents a clinical risk has not been reached. We hope that this report on a unique manifestation of HFI will help clinicians in evaluating patients with this condition.

## Introduction

Hyperostosis frontalis interna (HFI) is a rare phenomenon characterized by bony overgrowths involving the frontal bone and positioned bilaterally to the superior sagittal sinus [1]. Specifically, a new diploë layer forms deep to the internal table along with instances of mainly trabecular and lamellar bone [2]. Although the cause of HFI is currently not agreed upon among researchers, some leading theories involve hormone irregularities and changes in diet [2,3]. While HFI is typically viewed as a benign condition, associated symptoms such as frontal lobe dysfunctions lead to questions about its clinical relevance [3,4]. HFI is more prevalent in postmenopausal females [3]. Our case presents a possible novel growth pattern characterized by cancellous bone crossing the superior sagittal sinus, a feature not explored in the literature. Grading of HFI can be utilized to model the growth patterns first presented by Hershkovitz et al. The grading scale involves increasing levels of severity/degrees of growth, traditionally from A to D [1]. Recently, hyperostotic involvement of the falx cerebri has been considered class E HFI [5]. Except for the superior sagittal sinus growth, this specimen fits clearly into the C category due to the excessive elevation and coalescence of the pathological bone in the frontal region.

## Case presentation

**Figure 1:**
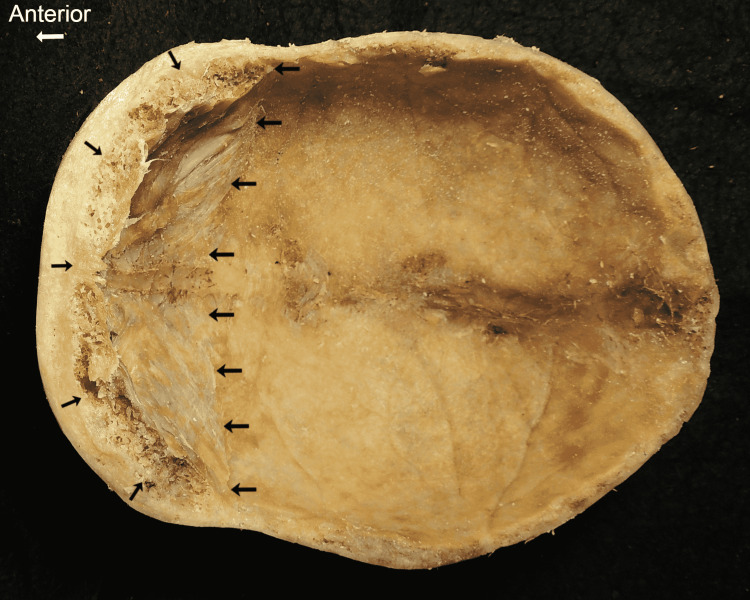
Arrows indicate boundaries of growth. Asymmetry in thickness of growth is noted when comparing the left and right sides

HFI was found in an 89-year-old woman during a routine anatomy dissection course at the Lake Erie College of Osteopathic Medicine in Bradenton. A transverse cut of the calvaria revealed a buildup of the trabecular bone deep to the internal table of the frontal bone (Figure 1). Visible erosion of the frontal lobe of the brain was noted after removal. However, it remains unclear whether the damage was caused by the physical removal of the calvaria, or whether the abnormal growth was to blame. The osseous thickening spanned approximately 1.3 cm into the cranial cavity. Interestingly, slight growth was noted on the lesser wing of the sphenoid bone. In terms of the margins, the bony nodules tapered to a smoother pattern and had a sharp demarcating edge anterior to the middle meningeal groove (Figure 1).

**Figure 2:**
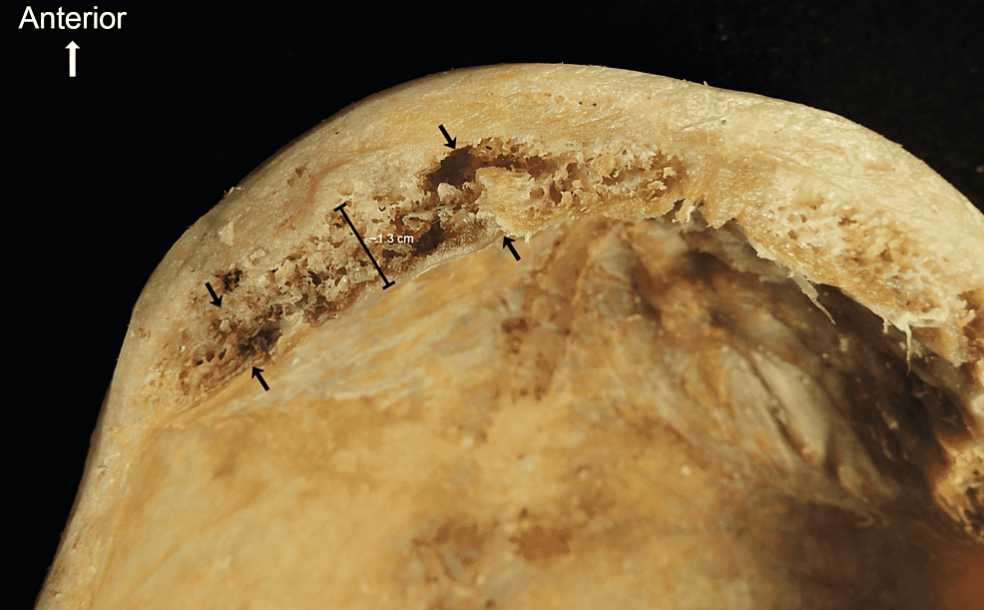
Trabecular bone growth measures approximately 1.3 cm at the labeled point. Arrows demonstrate growth confined deep to the internal table of the frontal bone

The trabecular bone growth seen in Figure 2 arose from deep to the internal table of the frontal bone. The labeled area in Figure 2 measured approximately 1.3 cm. The growth thickens the more lateral it is from the superior sagittal sinus.

**Figure 3:**
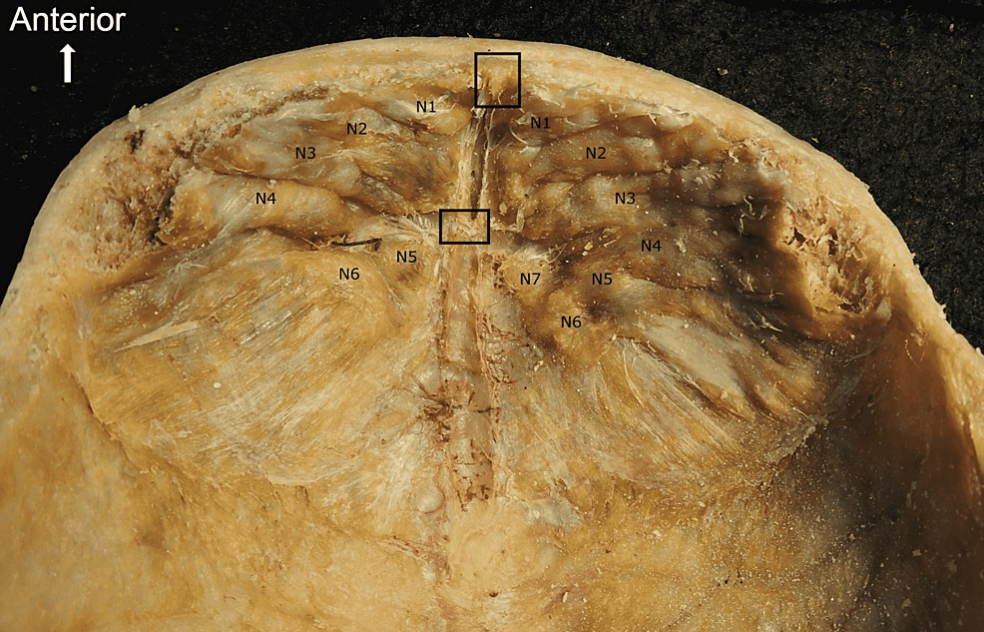
Black rectangles indicate osseous thickening at the superior sagittal sinus. Distinct nodules are numbered (N1, N2, etc.) on each side of the superior sagittal sinus

The growth exhibited a continuous curve deep to the internal table of the frontal bone and notably included osseous thickening at the superior sagittal sinus as seen in Figure 3. Additionally, the growth was coupled with distinct nodule formation on each side. The nodules were characterized as noncontiguous and bilaterally asymmetric. Bony nodules were more prevalent and numerous on the left side of the frontal bone as seen in Figure 3. Based on the given history, our cadaver had dementia and atherosclerosis. Whether HFI was the cause of these conditions is unclear. The cadaver did not have any other significant abnormalities.

## Discussion

HFI is a condition involving abnormal growth of the internal table of the frontal bone. The growth can involve levels of nodular growth and at times accompany neurological symptoms [6]. The etiology and possible pathology of HFI are not clinically agreed upon; however, it predominantly affects postmenopausal women, indicating a possible endocrine cause [1,3]. According to the classification used most widely for diagnosing HFI and first introduced by Hershkovitz et al., our case represents a class C HFI [1]. Nevertheless, this present case may demonstrate novel growth by showing an invasion of spongy bone into the superior sagittal sinus, a feature not described in the literature. Hershkovitz et al. presented a robust grading system for HFI in cadavers [1]. Clinical grading scales do exist, but they are not nearly as robust as the classification system used in cadavers. For instance, a three-scale system was proposed based on volume-rendering diagnostic imaging [7]. There were clear limitations in identifying the early stages of HFI with reasonable accuracy [7]. Considering the symptoms associated with HFI, perhaps a grading scale linking symptoms with degrees of bone growth can be established.

Recent studies on HFI undoubtedly suggest an increasing prevalence of the condition [3,8,9]. The risk of developing HFI has also increased by a factor of 2.5 with more severe cases arising in younger age populations compared to older generations [8]. The frequency of HFI in the general population is believed to be between 5 and 12% depending on factors such as age and sex [3]. Reports demonstrate a higher risk of HFI in postmenopausal females; Vuković et al. suggest a 16.4% prevalence among postmenopausal females [9]. Perhaps, additional diagnostic measures should be taken considering the increasing prevalence of HFI.

HFI is commonly found incidentally during routine clinical diagnostic imaging of the skull [4]. However, due to the conflicting theories regarding HFI’s clinical significance, it is rarely searched for as a pathological explanation for clinical symptoms [10]. Now, with an increasing prevalence of the disease and more recent research on the topic, further discussion is warranted. Histological research has found cadaveric cases of HFI presenting with a layer of cortical bone along with typical spongy bone [2]. It is possible that HFI cases with layers of cortical bone will present with more significant increases in intracranial pressure and lead to more severe symptoms. These HFI-associated symptoms may present as frontal headaches, seizures, lobar atrophy, or memory loss [6,8,4]. The literature also associates HFI with dementia, as is the case with our specimen [10]. Additional pathologies associated with HFI include metabolic and endocrine disorders such as Morgagni-Stewart-Morel (MSM) syndrome, which has a strong genetic basis [2,6]. Despite these clinical correlations, HFI is still generally an incidental finding and classified as a benign condition [3].

Although there is still uncertainty, endocrine abnormalities are a strong hypothesis for the cause of HFI. Postmenopausal females are more likely to develop the condition. Therefore, estrogen dysregulation may play an important role in HFI. According to a recent histological analysis, HFI growth resembles that of a new diploë layer of the frontal bone [2]. This “diploization” is characterized by highly vascular networks that promote osteogenesis. Estrogen is known as a key regulator of meningeal microvascular networks and thus may explain the higher risk in postmenopausal populations [11,12]. A metabolic component may further explain the etiology of HFI. Modern women have been exposed to vastly different diets and lifestyles following the Industrial Revolution. This period coincides with the increase in the prevalence of observed HFI over the last century [1,2,13].

## Conclusions

HFI mainly presents with spongy bone growth deep to the internal table of the frontal bone. Our case had asymmetrical nodular thickening and possible novel superior sagittal sinus growth. Robust cadaveric HFI classifications from A to E have been established, with our specimen falling into the class C category. Currently, clinical grading scales of HFI have clear limitations. This coincides with the lack of agreement as to the clinical significance of HFI. This report documented a unique case of HFI and discussed its ever-increasing prevalence.
